# Impact of Particle Irradiation on the Immune System: From the Clinic to Mars

**DOI:** 10.3389/fimmu.2017.00177

**Published:** 2017-02-22

**Authors:** Rodrigo Fernandez-Gonzalo, Sarah Baatout, Marjan Moreels

**Affiliations:** ^1^Radiobiology Unit, Laboratory of Molecular and Cellular Biology, Institute for Environment, Health and Safety, Belgian Nuclear Research Centre, SCK-CEN, Mol, Belgium

**Keywords:** protons, carbon ions, immunotherapy, space flight, cosmic radiation, immune response, cancer therapy

## Abstract

Despite the generalized use of photon-based radiation (i.e., gamma rays and X-rays) to treat different cancer types, particle radiotherapy (i.e., protons and carbon ions) is becoming a popular, and more effective tool to treat specific tumors due to the improved physical properties and biological effectiveness. Current scientific evidence indicates that conventional radiation therapy affects the tumor immunological profile in a particular manner, which in turn, might induce beneficial effects both at local and systemic (i.e., abscopal effects) levels. The interaction between radiotherapy and the immune system is being explored to combine immune and radiation (including particles) treatments, which in many cases have a greater clinical effect than any of the therapies alone. Contrary to localized, clinical irradiation, astronauts are exposed to whole body, chronic cosmic radiation, where protons and heavy ions are an important component. The effects of this extreme environment during long periods of time, e.g., a potential mission to Mars, will have an impact on the immune system that could jeopardize the health of the astronauts, hence the success of the mission. To this background, the purpose of this mini review is to briefly present the current knowledge in local and systemic immune alterations triggered by particle irradiation and to propose new lines of future research. Immune effects induced by particle radiation relevant to clinical applications will be covered, together with examples of combined radiotherapy and immunotherapy. Then, the focus will move to outer space, where the immune system alterations induced by cosmic radiation during spaceflight will be discussed.

## Introduction

The main purpose of radiotherapy is to induce DNA damage in tumor cells to trigger a network of events leading to cell death. However, conventional photon radiotherapy can also modify the tumor phenotype and its environment, making the tumor more vulnerable to the immune system. Some examples are the increased expression of major histocompatibility complex I in tumor cells (1) and the modulation of *in situ* secretion of cytokines (2). In addition to local alterations, it is now obvious that the effect of radiation goes beyond the cells directly affected by the irradiation source, leading to non-targeted effects. Indeed, several studies have shown that radiation exposure is harmful for non-irradiated cells (3, 4). However, it is also clear that non-targeted radiation effects can lead to abscopal effects, i.e., an uncommon event of tumor regression at a site or tissue distant from the primary site of radiation (5). Case reports showing remission of metastatic events in specific conditions are believed to be the result of abscopal effects (6–8). Although the information regarding the driving machinery controlling these abscopal effects is not totally understood, the immune system plays a key role (9–11). This information highlights the importance of antitumor immunity development after radiation therapy, and the potential added benefits of combined radiotherapy and immunotherapy.

Most of the data about radiation-induced immune alterations are based on research employing photon-based sources (i.e., gamma rays and X-rays). Although conventional radiation is the most popular tool used in clinical settings, antitumor treatments using particle irradiation (i.e., protons or carbon ions) are becoming a useful alternative. Indeed, the number of facilities allowing for particle therapy is growing at a high rate in the last decade.^1^ From a physical perspective, the inverted depth-dose profile and the sharp dose fall-off after the Bragg peak offered by particle beams allow for a more precise localization of the radiation dosage to the tumor, as compared to conventional photons (12). Besides the ballistic advantage, the use of high-linear energy transfer (LET) carbon ion beams offers a biological advantage as well, i.e., a higher relative biological effectiveness (RBE) compared to photon and proton exposure (13). However, recent studies suggest that both protons and carbon ions have unique biological properties when compared with photons (14, 15). Considering these physical and biological characteristics, different radiation types could induce divergent immune alterations, which will in turn determine the type and magnitude of local and systemic (abscopal) immune effects.

Contrary to local irradiation for tumor treatment, there are some particular situations where the entire human body is subjected to particle irradiation, such as human space flight. In this case, healthy tissue will be chronically exposed to energetic radiation, inducing different effects on the immune system (for a comparison, see Table 1). Although the immune alterations during space flight are mainly a result of microgravity, psychological stress, and radiation (16), it is believed that radiation *per se* will be a major determinant of astronauts’ immune system status in long-term, exploratory-type missions. Supporting this hypothesis, there are several animal studies showing how simulated cosmic radiation clearly interferes with the immune system (see below).

**Table 1:** **Radiation characteristics of particle therapy and cosmic radiation and effects on the immune system**.

	Particle therapy	Cosmic radiation
Target tissue	Very localized → tumor	Whole body, healthy tissue
Exposure	Several sessions (fractionation)	Chronic
Source	Particle accelerators	Galaxy, the Sun
Particle type	Protons or heavy ions such as C (also H, Li, O, etc.)	Protons, H ions, HZE particles (e.g., Fe), and electrons
Maximal energy	~200 MeV for protons and ~600 MeV for C ions	~1,000 MeV for protons and ~600 MeV for heavy ions
Dose	Target tissue: high – 60–80 Gy-eq	~662 mSv (in a round trip to Mars, without considering the time spent in the planet)
Secondary irradiation	Problem of neutrons leakage	Secondary cosmic rays due to vehicle shielding
Short-term immune effects	Alterations in tumor cells and their environment leading to immunogenic cell death	^a^Significant downregulation of different components of the immune system [more affected—B cell > T cells (CD8+ > CD4+) > NK cells—less affected]
Long-term immune effects	Systemic immunogenic response affecting specific tumor cells both local and distant (abscopal effects)	^a^Mainly unknown. Immune system downregulated response may persist
Potential outcome	Improve survival of cancer patients	Immune dysregulation → major health risk for astronauts during exploratory missions

*H, hydrogen; C, carbon; Li, lithium; O, oxygen; Fe, iron; NK, natural killer*.

*^a^From animal studies*.

The purpose of this mini review is to briefly present the current knowledge in immune alterations triggered by particle irradiation and to propose new lines of future research. In particular, immune effects induced by particle radiotherapy (protons and carbon ions) will be covered, together with promising examples of combined particle therapy and immunotherapy. Then, we will focus on a very specific situation where healthy humans are subjected to this type of radiation (i.e., space flights), and how such environment might affect their immune system.

## Particle Therapy

Radiotherapy is used for local tumor control through radiation-induced killing of cancer cells. Nowadays, it is accepted that the success of radiotherapy also involves the immune system. The radiation-induced tumor cell death enables the presentation of tumor-derived antigens by dendritic cells (DCs). These antigen-presenting cells function as a bridge between the innate and adaptive immunity (17), thereby leading to the induction of tumor-specific cytotoxic T cells (18–20).This can result in the initiation of local and systemic antitumor immune responses (10). In this regard, adequate combination of radiotherapy and immunotherapy has opened new possibilities to treat metastatic and advanced cases. Common immunotherapeutic strategies combined with radiation include administration of cancer-specific antibodies, cytokines, cancer vaccines, and immune checkpoint inhibitors (21).

Besides conventional radiotherapy, particle therapy with protons or carbon ions has become the treatment of choice for specific cancer types. Due to the improved dose distribution, particle irradiation enhances local tumor control. In addition, both proton and carbon ions show unique molecular and cellular responses compared to photon radiation (14, 15, 22).

### Preclinical Studies

Conventional radiotherapy increases the expression of specific surface molecules, adhesion molecules, death receptors, stress-induced ligands, and classical stimulatory molecules, leading to increased immunogenicity (23). However, only a limited number of preclinical *in vitro* and *in vivo* studies have investigated whether particle radiation can modify tumor cells into a more immunogenic phenotype, increasing their sensitivity to immune surveillance.

Proton irradiation modulates several processes critical in tumor advancement and progression, including angiogenesis and immunogenicity. Indeed, decreased levels of vascular endothelial growth factor, interleukin (IL)-6, IL-8, and hypoxia-inducible factor 1-alpha, have been reported in lung carcinoma cells after proton exposure (14, 22). A recent study reported how sublethal proton or photon irradiation induced a similar increase in the levels of surface molecules involved in T-cell recognition, as well as translocation of calreticulin to the tumor cell surface (24). The latter is critical for increased sensitivity to T cell killing (25). These changes in the immunogenic phenotype in a wide array of cancer cell types after proton exposure can make malignant cells more sensitive to T-cell-mediated cell death. In addition, proton therapy induced a downregulation of PD-L1 in prostate tumor cells, potentially leading to a higher T-cell activity in irradiated tumors (24).

Our group has shown that carbon ion irradiation induces changes in the expression of genes involved in tumor progression in human prostate cancer cells (26). Genes involved in cell migration and motility were expressed in a dose- and time-dependent manner (27). Recently, the immunogenic alterations induced by carbon ion irradiation were investigated in another human *in vitro* model (28). Carbon ion radiation increased the levels of high mobility group box 1 (HMGB1) in the culture supernatants of different human cancer cell lines. HMGB1 plays an important role in antigen-presenting cell activation and induction of an efficient immune response (10, 29). The levels of HMGB1 induced by carbon ion exposure were comparable with iso-survival doses of X-rays, and the applied doses were similar to those used in the clinic.

Surprisingly, although the number of patients treated with protons far exceeds that of carbon ion-treated patients, preclinical studies with particles have focused on the impact of carbon ion irradiation on the antitumor immunity. Preclinical *in vivo* studies using carbon ion radiation have convincingly demonstrated that carbon ion exposure induces antitumor immunity in immunocompetent animals, together with abscopal effects in some cases. When compared with photon irradiation, carbon ion exposure reduced the number of distant lung metastasis in carcinoma models in immunocompetent mice (30) and induced a higher expression of membrane-associated immunogenic molecules in mice tumors (31). The latter may highlight the superior potential of particle therapy vs. photons to be combined with immunotherapy. On the contrary, no greater benefits of carbon ions compared with photons were observed in a pulmonary metastatic murine model (32).

To our knowledge, only two preclinical studies combining carbon ion radiation and immunotherapy have been published (33, 34). In both cases, carbon ion irradiation was combined with the administration of bone marrow DCs in immunocompetent mice. Although carbon ion exposure alone had enough potential to activate DCs (33), greater antitumor immunity and a reduction in the number of metastasis were reported after combined radiotherapy and immunotherapy (33, 34). Interestingly, the combination of DC immunotherapy and photon radiation was not able to induce the same effects (35). These results suggest that even when exposed to the same equivalent doses, carbon ion therapy might activate the immune system to a greater extent than conventional radiotherapy.

### Case Reports and Clinical Trials: Immune-Activating Properties of Radiotherapy

Abscopal responses after particle therapy have been occasionally reported in patients treated in Japan. A patient with colon carcinoma and distant lymph node metastasis was treated with local carbon ion therapy. Six months after treatment, both the primary tumor and the metastasis resolved (36). Abscopal regression was also described in a patient who had both abdominal and para-aortic lymph node metastases. He was treated with different fractions of carbon ion irradiation for the abdominal lymph nodes only. After 6 months, both metastases were reduced (37).

With the increase in knowledge about the combination of particle radiotherapy and immunotherapy, novel clinical trials employing particle therapy with adjuvant systemic immunotherapy must be performed. Indeed, several clinical trials investigating such combination of therapies are currently ongoing,^2^ and some have already been completed. The combination of intratumoral injection of hydroxyapatite as immune adjuvant and proton beam therapy was found to be feasible and safe in patients with locally advanced or recurrent hepatocellular carcinoma (phase I study) (38). Four of nine patients were progression free for >1 year.

### Open Questions

Dose and fractionation are likely to be key variables in determining the effects of ionizing radiation on the immune system of the patients and/or in determining the success of radiotherapy when combined with different forms of immunotherapy. Indeed, modifying the fractionation protocol can have a major impact on, e.g., DCs, activation (39). In the context of particle therapy, enhanced immune reactions may be involved in response to hypofractionation, where patients receive a higher single dose in a lower number of fractions (37). Until now, data describing the immunogenicity in response to particle therapy are mostly based on single high-radiation doses, which are not commonly used in clinical settings. In addition, when analyzing combined approaches, the correct sequencing of radiation may depend on the type of immunotherapy chosen. Therefore, there is an urgent need to test and further identify the immunogenic properties of different fractionation schemes, and how such schemes should be combined with immunotherapy to boost beneficial adaptations. In addition, proton treatment is now based on a generic RBE value of 1.1. However, when treating a tumor volume, the RBE is increasing in the distal end of the spread-out Bragg peak (40). Research is needed to investigate whether different RBEs of protons affect the immunogenicity in different parts of the tumor.

## Space Radiation

Cosmic radiation is composed of galactic cosmic rays (GCRs) and solar particle events (SPEs) (41). GCRs are the continuous, background radiation that any mission beyond the magnetic field of the Earth will encounter. Protons are the main component of the GCRs, making up to 85% of the total. About 11–14% of the GCRs flux is helium ions, and ~2% consist of electrons. Importantly, there is a small, still very significant 1% of high (H) atomic number (Z) and high-energy (E) particles (HZE particles). Among them, Iron is the most important one due to its high LET. In addition to the GCRs, astronauts undergoing interplanetary missions will have to face the unpredictable SPEs containing energetic particles (mainly protons) from the Sun. Of note, the shielding used in the spaceship can interact with particles from GCRs and SPEs creating secondary radiation, which can increase the effectiveness of irradiation by heavy ions (42). All in all, the most accurate estimations indicate that the radiation levels for the shortest round-trip to Mars would be in the order of >0.6 Sv (43), which is close to, or even above, the dose limits proposed by NASA for the entire career of an astronaut (44). Indeed, radiation is one of the major concerns to maintain astronauts’ health during exploratory-type missions.

The adverse effect of cosmic radiation on the immune system is a matter of concern, given that some components of the immune system are among the most radiation-sensitive tissues in the body (41). A good example is the chromosomal aberrations described after missions in low-Earth orbit [e.g., Ref. (45–49)], where the dose is much lower than that expected during a trip to Mars. When exposed to simulated cosmic radiation with or without shielding, *in vitro* human lymphocytes also show chromosomal damage, which is dependent on the radiation quality, and the type of shielding (50, 51).

Despite investigations performed in low-Earth-orbit, there is a lack of data analyzing the effects of cosmic radiation beyond Earth’s magnetic fields on the human immune system. Indeed, only a few humans have gone far enough to experience the full spectrum of cosmic radiation (NASA Apollo missions). Hence, the most appropriate approach we currently have to study cosmic radiation-induced effects on the immune system are animal models. The research team of Drs. Griedly and Pecaut has performed multiple ground-based experiments with murine models to analyze the immune system response to simulated GCRs and SPEs. These investigations have shown that simulated cosmic radiation decreases the lymphocyte population (52), with a higher impact on B cells, followed by T cells (CD8+ > CD4+) and natural killer cells (53–55). The response and function of leukocytes are also altered by simulated cosmic radiation (55), and the effects are long lasting and dependent on the dose, the type of source, and the body compartment (56–59). Overall, these results are supported by investigations from other groups (60, 61).

A matter of debate regarding cosmic radiation is whether previous radiation exposure can make humans more radioresistant. Pre-exposure to low-dose photon radiation may decrease the regeneration capacity of lymphocytes after simulated SPE radiation (62). Contrarily, previous exposure to low-dose radiation may offer some degree of radioprotection to reduce expansion of T regulatory cells (Treg; immunosupressive cells involved in tumor development) in response to subsequent proton (e.g., SPE) irradiation (63). This is important given that the proportion of Treg cells is increased after simulated SPE radiation (62). Despite the controversy about potential radioprotection from pre-exposure with low-dose radiation to SPEs or GCRs at the cell pupulation level, it seems clear that such priming technique modifies the molecular signaling of immune cells in mice (64, 65).

### Open Questions

In addition to the uncertainties and difficulties to study the immune system alterations to space radiation, it should be noted that radiation is not the only “space stressor” affecting the immune system. Confinement, circadian rhythms, psychological stress, and microgravity may add (or counteract) the effects of radiation in the immune system of astronauts (16). To study the combined effects of multiple space stressors, *in vitro* models should provide a valuable platform (66). Indeed, controversy exists about whether microgravity increases human immune system radiosensitivity in space, by, e.g., increasing cell apoptosis after heavy ion exposure (67, 68) or not (69). In addition, better models and platforms allowing investigations using human living tissues and space-like radiation should be developed. Finally, an interesting and ongoing topic of research valuable for future long-term space missions is the development of tools to identify the subjects that are more resistant to space radiation.

## Concluding Remarks

From a physical point of view, the rationale for the use of particle irradiation in cancer therapy has been obvious for a very long time. Adding to this, recent studies have shown that particle therapy may exert interesting effects on tumor cells that might eventually increase the effectiveness of the antitumor immune response.

Surprisingly, there is a lack of preclinical *in vivo* data combining proton therapy and immunotherapy, and the number of preclinical studies with carbon ions is very limited. This topic warrants further investigation to exploit this strategy to kill cancer cells that are outside the primary radiation field. Promising approaches include the combination of particle therapy with immune system modulators, immune checkpoint inhibitors, and cytokines.

Besides clinical studies, basic research should investigate underlying immune mechanisms leading to an efficient antitumor response. This research may translate into new, more effective therapeutic approaches. Moreover, differences in individual radiosensitivity, which may influence non-targeted, abscopal radiation responses, should be further examined.

Individual radiosensitivity should also be analyzed in the context of human spaceflight and immune system alterations, together with the potential beneficial effects of pre-exposure to low-dose radiation before spaceflight. Given that cosmic radiation clearly downregulates the immune system, such information could help selecting the most appropriate individuals to succeed in a long-term, exploratory mission.

## Author Contributions

RF-G and MM: conception, drafting, and revising of the review article. SB: conception and revising of the review article.

## Conflict of Interest Statement

The authors declare that the research was conducted in the absence of any commercial or financial relationships that could be construed as a potential conflict of interest.

## Footnotes

^1^https://www.ptcog.ch/.

^2^https://clinicaltrials.gov/.
